# Delayed seropositivity is associated with lower levels of SARS-CoV-2 antibody levels in patients with mild to moderate COVID-19

**DOI:** 10.1186/s42506-023-00131-x

**Published:** 2023-03-21

**Authors:** Marwa M. Fekry, Hanan Soliman, Mona H. Hashish, Heba S. Selim, Nermin A. Osman, Eman A. Omran

**Affiliations:** 1 Microbiology Department, High Institute of Public Health, Alexandria University, Alexandria, Egypt; 2 Ministry of Health and Population, Cairo, Egypt; 3 Biomedical Informatics and Medical Statistics Department, Medical Research Institute, Alexandria University, Alexandria, Egypt

**Keywords:** Seroconversion, Anti-spike, Anti-nucleocapsid, SARS-CoV-2

## Abstract

**Background:**

Patients with COVID-19 can develop a range of immune responses, including variations in the onset and magnitude of antibody formation. The aim of this study was to investigate whether SARS-CoV-2 antibody levels vary in patients with mild to moderate COVID-19 in relation to the onset (days) of their post-symptom seropositivity and to explore host factors that may affect antibody production

**Methods:**

This was a prospective, multiple measurements study involving 92 PCR-confirmed patients with mild to moderate COVID-19. Antibody testing for anti-nucleocapsid (anti-NP) and spike proteins (anti-S) was performed using ELISA tests. Serum samples were collected over a period of 55 days from symptom onset of COVID-19 infection, and repeated as necessary until they turned positive.

**Results:**

No significant differences were found between the positivity rates of anti-S or anti-NP regarding any clinical symptom (*p* > 0.05). The majority of patients who tested positive for anti-NP and anti-S showed early seropositivity (within 15 days of symptom onset) (75.9% for anti-NP and 82.6% for anti-S). Younger patients, those without chronic diseases, and non-healthcare workers had the highest percentage of seroconversion after day 35 post-symptom onset (*p* = 0.002, 0.028, and 0.036, respectively), while older patients and those with chronic diseases had earlier seropositivity and higher anti-NP levels (*p* = 0.003 and 0.06, respectively). Significantly higher anti-S ratios were found among older (*p* = 0.004), male (*p* = 0.015), and anemic patients (*p* = 0.02). A significant correlation was found between both antibodies (*p* = 0.001). At the end of the study, the cumulative seroconversion rate for both antibodies was almost 99%.

**Conclusions:**

Some COVID-19 patients may exhibit delayed and weak immune responses, while elderly, anemic patients and those with chronic diseases may show earlier and higher antibody responses.

**Supplementary Information:**

The online version contains supplementary material available at 10.1186/s42506-023-00131-x.

## Introduction

In December 2019, an outbreak of coronavirus infectious disease (COVID-19), caused by the severe acute respiratory syndrome coronavirus 2 (SARS-CoV-2), became a pandemic with a significant public health impact [1]. Patients with mild COVID-19 typically experience symptoms such as fever, fatigue, sore throat, cough, and others, but no lower respiratory symptoms. In contrast, patients with moderate COVID-19 exhibit additional evidence of pneumonia but their oxygen saturation is still ≥ 94% on ambient air [1, 2]. Due to limited medical resources, a large number of suspected or confirmed mild-to-moderate COVID-19 patients are not hospitalized, and are instead isolated and treated at home [3].

SARS-CoV-2 contains several immunogens, importantly, the spike (S), and nucleocapsid protein (NP). Antibodies against NP indicate previous exposure to the virus and are highly abundant, sensitive, and immunogenic. Anti-S immunoglobulins (IgGs) reflect the immune status and correlate well with neutralizing antibodies [4, 5].

Two opposing theories have been proposed regarding the role of antibodies in viral infections. The first theory suggests that antibodies have a protective role in clearing the viral infection, while the second theory suggests they could lead to antibody-dependent enhancement and deterioration in clinical severity [6]. A systematic review of 150 studies reported that the IgG levels peak between weeks 3 and 7 post-symptom onset [7]. Reviews of the published literature indicate that more than 90% of patients develop IgG seropositivity following primary infection, with rates ranging between 91% and 99% in large studies [7, 8].

Monitoring anti-SARS-CoV-2 immune response can help predict the chances of re-infection. It is still not clear why some patients experience delayed or absent seropositivity following confirmed COVID-19 infection, and whether specific host factors contribute to this [9]. This study aimed to investigate the levels of SARS-CoV-2 antibodies in relation to the onset of seropositivity among mild-to-moderate COVID-19 patients. Additionally, factors associated with weaker and delayed seroconversion were studied.

## Methods

### Study design and sample size calculation

This prospective study involved repeated sample measurements within a 55-day period, through the period from June 2020 to December 2020 which coincided with the first and second waves of COVID-19 in Egypt. During this period, the predominant Pango lineages in Egypt were B, B.1, B.1.1, B.1.1.1, B.1.1.7 (alpha variant), B.1.170 and C.36 [10].

The sample size was calculated by assuming a seroconversion rate of 85% with a 10% error, an alpha of 0.05, and 80% power, resulting in a minimum required sample size of 75 patients [11]. To account for anticipated dropouts, the sample size was increased to 92. The sample size was calculated using G* power 3.1.9.6.

Patients were consecutively contacted and recruited through web-based invitations following their PCR-confirmed results until the required sample size was reached. A structured interview questionnaire sheet was designed and completed for each patient, including socio-demographic data, clinical data such as symptoms at the time of the first sample collection, and history of contact with COVID-19-positive patients. The results of chest computed tomography (CT) were recorded at the initial presentation.

Serum samples were collected at pre-defined intervals according to serostatus since the onset of COVID-19 symptoms: 15 days, 25 days, 35 days, 45 days, and 55 days. No samples were taken before “day 15” of symptom onset, as IgG antibodies require time to appear in serum. These time points were chosen to monitor the onset of seroconversion and its association with antibody titer. Samples showing positive results (seroconversion) for either antibody were not repeated, i.e., samples were repeated only when both antibodies showed negative results. Results of antibody levels and onset of post-symptom seroconversion were interpreted in relation to patient-associated risk factors such as age, gender, comorbidities, and others.

### Ethical considerations

Informed consent was obtained from each patient. The study was conducted in compliance with the Helsinki Declaration and was approved by the “Ethics Committee” of the High Institute of Public Health, Alexandria University. Participants were informed of their right to discontinue their participation in the study at any time without consequences. Anonymity and confidentiality were assured.

### Patient selection

The 92 enrolled patients were all adults who were home-isolated and confirmed by PCR to have had COVID-19 diagnosis, 15 days before our sample collection. All patients had mild-to-moderate COVID-19 infection and were treated and monitored by their physicians. All symptomatic patients reported that this was their first COVID-19 attack. Asymptomatic home-isolated close contacts were also included in our study. Asymptomatic individuals were defined as PCR-confirmed SARS-CoV-2 cases who did not exhibit any clinical symptoms including fever, upper respiratory symptoms, pneumonia, fatigue, and gastrointestinal symptoms at the time of testing and remained asymptomatic until the end of the study.

### Sample collection and processing

Blood samples of 3 ml were collected from each patient and divided into two portions. Two ml of blood were taken in EDTA tubes for measuring hemoglobin, white blood cell counts (WBCs), and lymphocytes. One milliliter of blood was kept in a plain tube for antibody detection. Blood samples collected for antibody detection were centrifuged at 3000 rpm, and the sera were separated and stored at − 20 °C until testing.

Antibodies against the viral nucleocapsid were detected using a commercially available electrochemiluminescence immunoassay kit “Elecsys anti-SARS-COV-2 kit (Roche, Basel, Switzerland) run on the Cobas® e411 automated platform (Roche Diagnostics). Serum samples were also tested for the detection of immunoglobulin class IgG against the S1 domain of the viral spike protein using the anti-SARS-CoV-2 sandwich enzyme-linked immunoassay technique (ELISA) (EuroImmun, Lübeck, Germany)

According to the manufacturer’s instructions, the results of the anti-NP test were interpreted as follows: values of the cut-off index (COI; signal sample/cut-off) < 1.0 were considered negative, while results ≥ 1.0 were considered positive. COI values were also recorded numerically (as recommended by several authors) [11, 12]. According to the manufacturer, the overall agreement of this anti-NP kit with a pseudo-viral neutralization test was found to be 87.0%, with a specificity of 99.80%, and a sensitivity of 99.5% (calculated at ≥ 14 days post-PCR confirmation).

According to the manufacturer’s instructions, the results of the anti-spike IgG ELISA test were evaluated semi-quantitatively by calculating the ratio of sample extinction to that of the calibrator. Results were interpreted as follows: ratios < 0.8 were negative, ratios ≥ 0.8–< 1.1 were borderline, and those ≥ 1.1 were considered positive. According to the manufacturer, the sensitivity of this test was 94.4% after 10 days from symptom onset, and its specificity was 99.6% [13].

Males were categorized as “anemic” if their hemoglobin level was < 13 g/dl, while females were considered anemic if their hemoglobin level < 12 g/dl. The normal lymphocytic count was considered to be 1000–4800 while lymphopenia was indicated at lower levels and lymphocytosis at levels > 4800 cells/cmm^3^. Normal WBC was considered to be 4000–11,000 cells/cmm^3^ [14].

In case of negative results for either antibody, serum samples were followed by repeated collection and measurement at 10-day intervals until seroconversion occurred or until the end of the 55 days decided for the end of the study. Laboratory and radiological results were only performed for the initial samples and were not repeated for subsequent samples.

### Statistical analysis

Statistical analyses were performed using IBM (SPSS) Statistics Version 24.0* software. Qualitative data were presented using frequency and percentage while quantitative data were described, and tests of significance were determined after checking normality using the Kolmogorov–Smirnov (K-S test) and Shapiro tests. The Mann-Whitney test and Kruskal-Wallis test were used to compare the variables between the two independent groups and more, respectively. The Friedmann test was used to compare the ratio/ COI among consecutive samples. A proportional *Z* test was conducted to compare single categories between two independent groups. The Spearman correlation test was used to test the association between quantitative parameters. The Kappa test was used to test the positive agreement between the studied antibodies. The McNemar-Bowker test was conducted to check the significant risk categories in determining the positivity of samples. A significance level was set below 5% [15].

Since laboratory and radiological test results were only done at initial presentation (first sample only), all statistical associations and correlations between laboratory results/clinical symptoms with antibody levels were only analyzed for the antibody results of the first samples.

## Results

Of the 92 COVID-19 patients who were home-isolated, the majority (70.7%) were between the ages of 30 and 59 years, while 16.3% were over 60 years old. Females were predominant (59.8%), including two pregnant women. The majority of participants (38%) did not work, while 34.8% were healthcare workers and the remaining 27.2% had other occupations. Almost half of the patients (51.1%) did not have any chronic disease, while 16.3% had hypertension and 23.9% had more than one chronic disease. A history of contact with a confirmed COVID-19 case was reported in 60.6% of patients, while 23.9% reported having a confirmed COVID-19 case in their households. The majority of patients (73.9%) had pneumonia as evidenced by chest CT.

Fever was the most common symptom among patients (85.9%), followed by respiratory symptoms (69.6%), myalgia, and arthralgia (46.7% %). The rates of loss of taste and smell and the presence of gastrointestinal manifestations (nausea, vomiting, and diarrhea) were comparable (40.2% and 34.8%, respectively). Sore throat, sneezing, rhinorrhoea had a similar prevalence among these patients (27.2%, 3.3%, and 3.3%, respectively).

The median (IQR) of WBCs was 4150 (1787) cells/cmm^3^, and for hemoglobin was 12.85 (1.60) g/dl, while the median lymphocytic count was 1320 (580) cells/cmm^3^.

Three individuals remained asymptomatic until the end of the 55-day study period despite having close contact with symptomatic cases. One of them was a 53-year-old female nurse with diabetes and a normal chest CT, who tested positive for both anti-NP and anti-S antibodies after 13–15 days. The second asymptomatic patient was a 54-year-old, male smoker, with pneumonia on CT, who tested positive for anti-NP at day 35 and for anti-S at day 25. The third asymptomatic patient was a 29-year-old male smoker, who had a positive anti-NP result at day 35 and a positive anti-S result at day 45.

All patients, except for two, achieved seroconversion within 55 days. One patient was seronegative for anti-NP and another one was seronegative for anti-S antibodies. Both patients refused further follow-up for their antibodies. An 18-year-old female with systemic lupus erythematosus and on immunosuppressive therapy showed seroconversion for both antibodies only at day 55. This patient had. Both pregnant women in our study showed seroconversion for both antibodies in their initial samples (day 15), and thus did not require further sample collection.

**Fig. 1:**
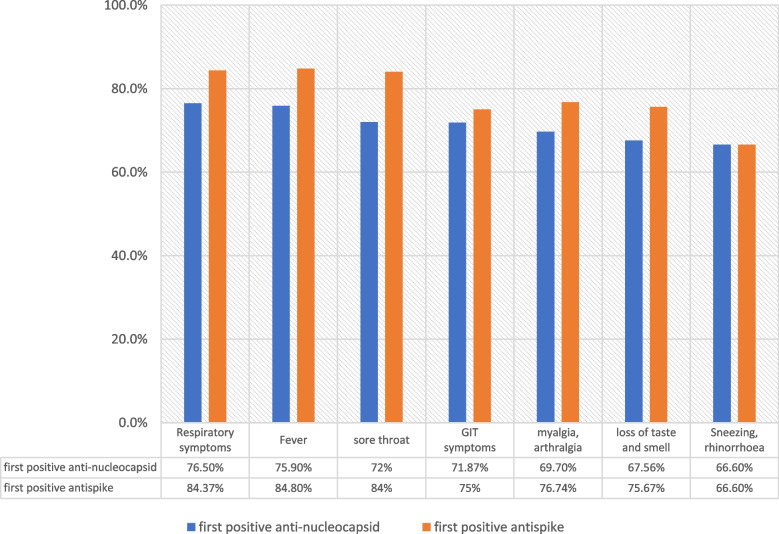
Clinical symptoms at initial presentation and antibody positivity (anti-NP and anti-S) of 92 patients with mild-moderate COVID-19

There was no significant difference in the positivity of either anti-S or anti-NP with any of the clinical symptoms (*p* > 0.05 using the *Z* test, data not shown). Overall, the anti-S was more seroprevalent in patients with any the symptoms (except sneezing and rhinorrhea) compared to the anti-NP. Fever, respiratory symptoms, and sore throat had the highest seropositivity of anti-S (ranging between 84% and 84.8%) (Fig. 1).

Pairwise comparison showed a significant difference between the titers of the samples showing seroconversion on days 15, 25, and 35. There was a significant difference (*p* = 0.03) in the median (min–max) COI values of anti-NP corresponding to the onset of seropositivity, which were as follows: 4.3 (0.08–140) on day 15, 2.23 (0.11–17.6) on day 25, and 1.54 (0.10–8.15) on day 35. There was a borderline significant difference (*p* = 0.05) between the median (min–max) ratios of anti-S, which seroconverted at different onsets: 3.39 (0.24–122) on day 15, 1.9 (0.5–4.5) on day 25, and 0.89 (0.45–1.11) on day 35. There was a significant difference (*p* = 0.01) between the results of the anti-NP and anti-S only in samples that seroconverted early (day 15), while this difference was not observed between both antibodies in samples that seroconverted on days 25 or 35 (probably due to their small sample size) (Table 1). The two samples showing seroconversion at days 45 and 55 were excluded from this table due to their statistical insignificance.

**Table 1:** Mean and median of SARS-CoV-2 anti-NP (COI) and anti-S (ratio) after 15, 25, and 35 days after onset of symptoms of COVID-19 among 92 home-isolated patients, Alexandria, Egypt

	Antibody level		*p* value
Days after onset of COVID-19 symptoms	COI of anti-NP	Ratio of anti-S	
15 days			
Mean ± SD	11.73 ± 20.01	8.8 ± 18.9	
Median (min–max)	4.3 (0.08–140)	3.39 (0.24–122)	0.01* ^b^
25 days			
Mean ± SD	4.1 ± 4.9	1.9 ± 1.15	0.23 ^b^
Median (min–max)	2.23 (0.11–17.6)	1.9 (0.5–4.5)	
35 days			
Mean ± SD	2.61 ± 2.94	0.83 ± .32	
Median (min–max)	1.54 (0.10–8.15)	0.89 (0.45–1.11)	0.31 ^b^
*P* value	0.03* ^a^	0.05 ^a^	

^a^*P < 0.05 (significant) using Friedman test

^b^*P < 0.05 (significant) using Wilcoxon signed rank test

Table 2 shows that there was no statistically significant difference between the seroconversion rates for both antibodies.

**Table 2:** Seroconversion of anti-NP and anti-S antibodies in relation to the days elapsed after the onset of symptoms of COVID-19 among 91 home-isolated patients, Alexandria, Egypt^a^

Days after onset of symptoms	Anti-NP		Anti-S		
	*N*	%	*N*	%	*p
15	69	75.9%	76	82.6%	0.283
25	16	17.6%	11	12.0%	0.413
35	5	5.4%	2	2.2%	0.459
45	0	0.0%	1	1.1%	0.999
55	1	1.1%	1	1.1%	0.477

^*^p < 0.05 (significant)

^a^One patient remained negative for anti-NP and another patient remained negative for anti-spike antibodies beyond 55 days after onset of symptoms and therefore they were not included in this table

There was substantial agreement between the results of anti-NP and anti-S antibodies (Kappa = 0.64 with *p* value = 0.0001). The agreement was calculated for the first samples only, as they constituted the majority of samples (69/92 samples were positive for anti-NP and 76/92 were positive for anti-spike). A significant correlation was found between both antibodies (*p* = 0.001). At the end of the study, the cumulative seroconversion rate for anti-NP and anti-S was 98.9% and 99%, respectively. Using the Spearman correlation test, a significant correlation was found between age and both anti-NP (*p* = 0.0001, *r* = 0.428) and anti-S (*p* = 0.003, *r* = 0.303). No statistical correlations were found between either of the antibodies or white blood cell count (WBCS), lymphocytic count, or hemoglobin (data not shown).

The majority of patients showed anti-NP and anti-S seroconversion in their first sample (15 days post-symptom onset). This was statistically significant for anti-NP regarding age, occupation, and the presence of chronic diseases (*p* = 0.02, 0.036, and 0.028 respectively). It was found that 73.4% of patients who were 30–59 years old, and all patients ≥ 60 years, showed anti-NP seroconversion after 15 days, compared to only 58.3% of patients < 30 years old. One-third of patients < 30 years old seroconverted for anti-NP after 35–55 days. Regarding occupation, healthcare workers (HCWs) showed anti-NP seroconversion in 80.6% of their samples after 15 days. This was in contrast to non-healthcare workers, who showed relatively later seroconversion (*p* = 0.036). It was also noticed that, among patients with chronic diseases, 86% of them showed anti-NP seroconversion on day 15, compared to 66.7% of patients without chronic diseases (Table 3).

**Table 3:** Demographic data and risk factors of 91 COVID-19 home-isolated patients according to the onset of positivity of SARS-CoV-2 anti-NP

Demographic data and risk factors	Days from symptom-onset till anti-NP positivity							*p* value
	15 days		25 days		≥ 35 days		Total	
	No.	%	No.	%	No	%		
Age categories(years)								
30	7	58.3	1	8.3	4	33.3	12	0.002*
30–59	47	73.4	15	23.5	2	3.1	64	
60+	15	100	0	0.0	0	0.0	15	
Sex								
Male	31	83.8	3	8.1	3	8.1	37	0.158
Female	38	70.3	13	24.1	3	5.6	54	
Occupation								
Not working	26	74.3	8	22.9	1	2.9	35	0.036*
Healthcare workers	25	80.6	6	19.4	0	0.0	31	
Non-healthcare workers	18	72	2	8	5	20	25	
Symptoms								
Asymptomatic	1	33.3	0	0.0	2	66.7	3	1
Symptomatic	68	77.3	16	18.2	6	4.5	88	
Chronic diseases								
No	32	66.7	10	20.8	6	12.5	48	0.028*
Yes	37	86	6	14	0	0	43	
Smoking								
Non-smoker	51	73.9	14	20.3	4	5.8	69	0.46
Smoker	18	81.8	2	9.1	2	9.1	22	
Contact with confirmed case								
No	28	77.8	6	16.7	2	5.5	36	1
Yes	41	74.5	10	18.2	4	7.3	55	
Confirmed household								
No	55	78.6	11	15.7	4	5.7	70	0.41
Yes	14	66.7	5	23.8	2	9.5	21	
Chest scan								
Not done	7	63.6	2	18.2	2	18.2	11	0.813
Normal	5	83.3	1	16.7	0	0	6	
Pneumonia	51	76.1	12	17.9	4	6	67	
Bronchitis	6	85.7	1	14.3	0	0	7	
Hemoglobin level								
Anemic	25	86.2	4	13.8	0	0	29	0.19
Non-anemic	44	71	12	19.4	6	9.6	62	
WBC count								
Less than 4000	30	75	7	17.5	3	7.5	40	0.86
Normal (4000–11,000)	38	76	9	18	3	6	50	
More than 11,000	1	100	0	0	0	0	1	
Lymphocytic level								
Lymphopenia	0	0	0	0	0	0	0	1
Normal	65	75.6	15	17.4	6	7	86	
Lymphocytosis	4	80	1	20	0	0	5	

^*^p < 0.05 (significant) using McNemar-Bowker test

There was a borderline significant association between age and seroconversion of anti-S (*p* = 0.051 (Table 4).

**Table 4:** Demographic data and risk factors of 91 COVID-19 home-isolated patients according to the onset of positivity of SARS-CoV-2 anti-S

Demographic data and risk factors	Days from symptom-onset till anti-S positivity							*p* value
	15 days		25 days		≥ 35 days		Total	
	No.	%	No.	%	No.	%	N	
Age categories								
< 30	10	83.3	0	0	2	16.7	12	0.051
30–59	51	79.7	11	17.2	2	3.1	64	
60+	15	100	0	0	0	0	15	
Sex								
Male	34	91.9	2	5.4	1	2.7	37	0.215
Female	42	77.8	9	16.6	3	5.6	54	
Occupation								
Not working	29	82.9	5	14.3	1	2.8	35	0.34
Healthcare workers	27	87.1	4	12.9	0	0	31	
Non-healthcare workers	20	80	2	8	3	12	25	
Symptoms								
Asymptomatic	1	33.3	1	33.3	1	33.3	3	1
Symptomatic	75	83.4	11	12.2	4	4.4	90	
Chronic diseases								
No	40	81.6	5	10.2	4	8.2	49	0.19
Yes	36	85.7	6	14.3	0	0	42	
Smoking								
Non-smoker	57	82.6	10	14.5	2	2.9	69	0.23
Smoker	19	86.4	1	4.5	2	9.1	22	
Contact with confirmed case								
No	33	91.7	3	83.3	0	0	36	0.16
Yes	43	78.2	8	14.6	4	7.2	55	
Confirmed household member								
No	61	88.4	6	8.7	2	2.9	69	0.08
Yes	15	68.2	5	22.7	2	9.1	22	
Chest scan								
No	7	63.6	1	9.1	3	27.3	11	0.06
Normal	5	83.3	1	16.7	0	0	6	
Pneumonia	58	85.3	9	13.2	1	1.5	68	
Bronchitis	6	100	0	0	0	0	6	
WBC count								
Less than 4000	33	82.5	4	10	3	7.5	40	0.53
Normal (4000–11,000)	42	84	7	14	1	2	50	
More than 11,000	1	100	0	0	0	0	1	
Hemoglobin level								
Anemic	27	90	2	6.7	1	3.3	30	0.82
Non-anemic	49	80.3	9	14.8	3	4.9	61	
Lymphocytic level								
Lymphopenia	0	0	0	0	0	0	0	0.6
Normal	72	83.7	10	11.6	4	4.7	86	
Lymphocytosis	4	80	1	20	0	0	5	

^*^p < 0.05 (significant) using McNemar-Bowker test

Older age was associated with higher values of anti-NP COI, with patients aged ≥ 60 years having the highest COI (median = 10.4), followed by those aged 30–59 years (median = 4.3), and then the youngest patients (< 30 years) (median = 2.5), *p* = 0.003). Significantly higher antibody ratios were also found among patients with chronic diseases (median (IQR) = 8.99 (22.9) compared to those without chronic diseases (median (IQR) = 3.40 (6.59), *p* = 0.006 (Supplement, Table S1).

Older age was associated with a higher anti-spike ratio, with patients aged 30–59 years, followed by those aged ≥ 60 years, having a significantly higher median (IQR) antibody ratio compared to younger patients (< 30 years) (ratios = 2.91 (4.6), 2.84 (7.9), and 2.28 (5), respectively, *p* = 0.004). Significantly higher antibody ratios were also found among males than females (ratio = 5.4 versus 2.4, respectively, *p* = 0.015). Anemia was also associated with higher anti-spike ratios compared to normal hemoglobin levels (ratio = 4.80 (9.98) *versus* 2.80 (4.76), *p* = 0.02) (Supplement, Table S2).

## Discussion

There are several uncertainties regarding the timing, quantity, kinetics, and persistence of SARS-CoV-2 antibody production. Although most individuals produce antibodies following infection, some patients either experience delayed seroconversion or do not generate an immune response at all [13, 16].

In our study, fever was the most common symptom among patients (85.9%), followed by respiratory symptoms (69.6%) and myalgia and arthralgia (46.7% %). Zhang et al. [17] reported that fever was the most common symptom (75.8%), while Guan et al. [18] reported a lower percentage of fever among similar patients (48.7%).

No statistical differences were observed between either of the two antibodies examined and any of the symptoms studied. In a similar study, fever and body ache were found to be correlated with higher antibody levels [19]. Differences between studies could be attributed to variations in disease severity, antibody kits used, and differences in sample collection.

The importance of asymptomatic infections lies in their ability to spread the infection in the community without taking precautionary measures, as they are not aware of their infectious state [4, 6]. A meta-analysis revealed that 17% of the total PCR-confirmed COVID-19 patients were asymptomatic [20]. In our study, only 3.3% of home-isolated patients were asymptomatic, which is comparable to another study reporting 2.1% of home-isolated patients without symptoms [13]. However, an Egyptian study among HCWs reported a much higher rate of asymptomatic cases, where anti-S seropositivity was found in 39.1% of unvaccinated participants. This might be due to their higher exposure to COVID-19 infection [21].

It is worth noting that one of our asymptomatic patients had pneumonia detected by CT, highlighting the importance of careful screening and follow-up of such cases. A meta-analysis reported that the asymptomatic rates were significantly lower among the elderly compared with children [13]. Another study found that asymptomatic COVID-19 persons were significantly more likely to be females and younger than symptomatic patients (38 versus 52 years) [16]. However, associated factors for the asymptomatic state could not be studied in our work due to the small numbers of asymptomatic participants. We observed that our three asymptomatic patients were seropositive at different time points. Only one asymptomatic patient in our study seroconverted early while the other two seroconverted more than 25 days after PCR positivity. The significance of delayed seroconversion in some patients is still unclear [22]. Unfortunately, in our study we could not assess the effect of viral load on symptomatic/asymptomatic states as we could not obtain data on viral load for all patients.

We found that the rate of seroconversion decreased with time, and late seroconverters also had a significantly lower antibody response compared to early-sero-converters**.** This finding is consistent with the results of Lucas et al., who also reported that antibody responses that develop within 14 days of symptom onset correlated with recovery, whereas those induced at later time points appear to lose this protective effect [23].

The time to seroconversion of anti-NP was significantly associated with age (*p* = 0.002), chronic diseases (*p* = 0.028), and occupation (*p* = 0.036) but not with sex. Additionally, the median anti-NP COI value, anti-S seropositivity, and ratio were all higher in the older age group indicating a stronger humoral response among older patients. This finding is consistent with the study by Amjadi et al. [19]. In contrast, other studies have reported an impaired antibody response with aging, attributed to B cell contraction and impaired antibody production following infections and vaccination [23]. Liu et al. did not find any associations between any demographic, clinical, and laboratory data with serostatus, although they reported a trend for increasing antibody positivity with increasing symptom severity [24].

In our study, we found significantly higher median anti-S ratios among males compared to females (*p* = 0.015). This finding is consistent with results reported by Amjadi et al. [19] who found that males had higher antibody levels and were also correlated with severe disease among hospitalized patients. This suggests that, males who are not hospitalized may be at greater risk of developing the severe disease compared to females.

In our work, it was observed that, older participant and those with anemia or chronic diseases had a higher and/or earlier immune response. Consistent with this finding, another study found that patients who tested positive for total antibodies were more likely to be diabetic or have an underlying malignancy than those who tested negative [18]. Amjadi et al. also reported in their study that greater disease severity, older age, male sex, and higher Charlson Comorbidity Index scores consistently correlated with higher antibody titers [19]. However, other reports suggest that older age and obesity may impair antibody responses [23, 25]. The contradictory results regarding differences in antibody production in relation to comorbidities warrant further investigations into their possible mechanisms. To better understand the determinants of immune response in COVID-19 patients and improve the early management of high-risk patients, more studies need to investigate the differences in risk factors. Long et al. [8] and Egger et al. [9] found a correlation between levels of COIs of anti-NP-positive patients and viral loads in their throat samples by PCR. They concluded that numeric values of this assay could be used to indicate the magnitude of antibody response. In our study, the mean value of COI of anti-NP was 11.73 ± 20.01, and the mean ratio of anti-S was 8.8 ± 18.9. The lower antibody levels in our study compared to those in other studies might be due to the difference in disease severity. Our study included only mild to moderate cases while other studies reported higher antibody levels in more severe COVID-19 patients [14].

In this study, we found that within 15 days after symptoms appeared a higher percentage of participants had positive anti-S compared to anti-NP (82.6% versus 75%). These results are consistent with the findings of Orth-Höller et al., who reported positive IgG titers in most mild and moderate patients after 2 to 3 weeks [26]. Similar to our findings, a study found that 1–3 months after symptoms, 98.3% of mild-moderate patients tested positive for anti-S compared to 85.6% for anti-N [27]. However, some researchers observed that anti-NP tend to appear earlier and are more sensitive than anti-S antibodies [5].

Our current study has shown a very high cumulative seropositivity rate (99%) by the end of the study period (55 days after symptom onset) which is consistent with previous studies [7, 8]. However, this is in contrast to the results of a study from Japan that reported anti-S seropositivity of only 47.8% in similar patients [6]. As our study has shown delayed or absent antibody production in some patients we recommend repeating negative antibody testing for clinically suspected patients who show seronegativity, until an average period of one month has elapsed. It is important to note that antibody testing results reflect a history of exposure rather than a recent infection diagnosis.

In our study, we found that two patients failed to seroconvert at the end of the 55 days. One patient remained anti-NP seronegative, while the other remained seronegative for anti-S. Other researchers have reported similar findings of serostatus suggesting that a small proportion of patients may have difficulty in rapidly developing immunity against SARS-CoV-2 and that their management needs to be more cautious as they may be prone to recurrence or re-infection [3]. It is also likely that seroconverters and non-seroconverters will probably also respond differently to vaccination. Recent studies revealed that seropositive persons have a heightened antibody response even after the first dose of vaccine, than those with weaker antibody responses. Additionally, COVID-19 patients who have been confirmed by PCR may be less inclined to get vaccinated, believing they are no longer at risk of infection. However, observations on the serostatus of infected persons in our study contradict this assumption [24].

Our results on seroconversion following infection by SARS-CoV-2 should be interpreted in the context of the prevailing viral variants in the country at the time of the study. Different viral variants can produce variable immune responses in terms of magnitude and neutralizing abilities. For instance, a study on B.1.1.298 variant showed a 10-fold lower antibody titer 24 h after inoculation compared to other SARS-CoV-2 strains [28]. Similar findings have been reported in studies on Omicron sub-lineages. The differences in antibody production can be attributed to antigenic differences between viral variants [29]. Moreover, it is expected that antibody responses will vary between different groups based on their characteristics and co-morbidities.

### Study strengths and limitations

Identification of factors associated with delayed seroconversion following infection can help in identifying individuals who are likely to show similar seronegativity after vaccination. In our study, clinical symptoms, and laboratory and radiological results were only recorded at the time of the first sample. So, they were studied in relation to the values of first tests for anti-S and anti-NP. A limitation of the study was that further samples were not studied with respect to the clinical condition of the patients. This should be considered in future studies. Association with PCR viral load could be also of value for correlation with antibody levels. Further studies with larger sample sizes are recommended to confirm of the reported risk factors, allowing the regression analysis model to identify predictors of weak and delayed seropositivity.

## Conclusions

The majority of mild-moderate COVID-19 patients showed seropositivity within 25 days following symptom onset. However, a minority of patients showed delayed seroconversion or did not convert at all. Early seroconverters were found to have a higher magnitude of antibody response when compared to delayed seroconverters, who had lower antibody levels. Host factors such as old age, chronic diseases, anemia, and male sex were found to be associated with early converters and those with heightened immune responses.

## Supplementary Information

**Additional** **file** **1: Table S1.** SARS-CoV-2 anti-NP (COI) in relation to demographic data and risk factors of 92 COVID-19 home-isolated patients. **Table S2.** SARS-CoV-2 anti-spike (ratio) in relation to demographic data and risk factors of 92 COVID-19 home-isolated patients.

## Data Availability

The datasets used and/or analyzed during the current study are available from the corresponding author on reasonable request.

## Abbreviations

COVID-19: Coronavirus infectious disease
SARS-CoV-2: Severe acute respiratory syndrome coronavirus 2
Anti-S: Anti-spike
Anti-NP: Anti-nucleocapsid protein
IgG: Immunoglobulin
CT: Computed tomography
COI: Cut-off index
