# Supplementary material for: Delayed seropositivity is associated with lower levels of SARS-CoV-2 antibody levels in patients with mild to moderate COVID-19
Source: J Egypt Public Health Assoc. 2023 Mar 21;98:6. doi: 10.1186/s42506-023-00131-x (PMC10027427; doi:10.1186/s42506-023-00131-x)
Supplement: Supplementary file 1 — Additional file 1: Table S1. SARS-CoV-2 anti-NP (COI) in relation to demographic data and risk factors of 92 COVID-19 home-isolated patients. Table S2. SARS-CoV-2 anti-spike (ratio) in relation to demographic data and risk factors of 92 COVID-19 home-isolated patients. [file 42506_2023_131_MOESM1_ESM.docx]

**Table S1: SARS-CoV-2 anti-NP (COI) in relation to demographic data and risk factors of 92 COVID-19 home-isolated patients**

| **Anti- NP (COI)**    **Demographic data and risk factors anti-NP ( COI)** | | | |
| --- | --- | --- | --- |
|  | | **Median (IQR)** | **P value** |
| **Age in years** | < 30 Yrs. | 2.5(4.05) | 0.003* |
|  | 30-59 | 4.3(12.07) |  |
|  | 60+ | 10.4(33.3) |  |
| **Sex** | Male | 6.7(11.5) | 0.114 |
|  | Female | 3.40(9.50) |  |
| **Occupation** | Not working | 3.43(10.16) | 0.205 |
|  | Healthcare workers | 7.25(17.59) |  |
|  | Non-healthcare workers | 3.5(7.9) |  |
| **Chronic diseases** | No | 3.40(6.59) | 0.006* |
|  | Yes | 8.99(22.9) |  |
| **Smoking** | Non-smoker | 4.2010.9) | 0.45 |
|  | Smoker | 6.7(11.4) |  |
| **Contact with confirmed case** | No | 6.03(16.40) | 0.33 |
|  | Yes | 3.60(9.6) |  |
| **Confirmed household member** | No | 5.25(12.48) | 0.15 |
|  | Yes | 3.29(7.13) |  |
| **Chest scan** | No | 3.83(9.98) | 0.92 |
|  | Normal | 5.86(27.34) |  |
|  | Pneumonia | 5.15(11.89) |  |
|  | Bronchitis | 3.91(24.15) |  |
| **Category of WBCs** | Leucopenia | 6.01(18.48) | 0.93 |
|  | Normal | 3.74(8.66) |  |
|  | leucocytosis | 13200 # |  |
| **Category of haemoglobin** | Anaemic | 5.76(11.65) | 0.36 |
|  | Normal | 3.68(12.34) |  |
| **Category of lymphocytes** | lymphopenia | No patient with<1000 | 0.42 |
|  | Normal | 4.34(10.16) |  |
|  | lymphocytosis | 12.75(26.84) |  |

** P < 0.05 (significant)* *# Only one patient had leucocytosis*

**Table S2: SARS-CoV-2 anti-spike (ratio) in relation to demographic data and risk factors of 92 COVID-19 home-isolated patients**

| **Demographic data and risk factors** | | **anti-S** [**ratio**](https://www.google.com/search?sxsrf=AOaemvLmwKxOywIAuBGVTmTvsWNhUS1JzA:1630809789494&q=quantitative&spell=1&sa=X&ved=2ahUKEwiYnMvx5-byAhXLAGMBHfg1D90QkeECKAB6BAgBEDA) | **p value** |
| --- | --- | --- | --- |
|  | | **Median (IQR)** |  |
| **Age ( years)** | < 30 | 2.28(5) | 0.004* |
|  | 30-59 | 2.91(4.6) |  |
|  | ≥60 | 2.84(7.9) |  |
| **Sex** | Males | 5.4(5.8) | 0.015* |
|  | Females | 2.4(4.5) |  |
| **Occupation** | Not working | 2.8(7.3) | 0.92 |
|  | Healthcare workers | 2.9(3.85) |  |
|  | Non-healthcare workers | 3.7(5.9) |  |
| **Chronic diseases** | No | 2.7(4.6) | 0.175 |
|  | Yes | 3.8(6.2) |  |
| **Smoking** | Non-smoker | 2.9(4.5) | 0.12 |
|  | Smoker | 5.4(7.4) |  |
| **Contact with confirmed case** | No | 4(6.1) | 0.078 |
|  | Yes | 2.7(4.9) |  |
| **Confirmed household member** | No | 3.7(5.6) | 0.097 |
|  | Yes | 1.9(5.3) |  |
| **Chest scan** | Not done | 3.9(10.5) | 0.34 |
|  | Normal | 2.3(3.1) |  |
|  | Pneumonia | 3.1(4.6) |  |
|  | Bronchitis | 7(32.39) |  |
| **Category of WBCs** | Leucopenia | 3.50(6.92) | 0.74 |
|  | Normal | 3.38(4.56) |  |
|  | Leucocytosis | 13200 # |  |
| **Category of haemoglobin** | Anaemic | 4.80(9.98) | 0.02* |
|  | Normal | 2.80(4.76) |  |
| **Category of lymphocytes** | lymphopenia | -- | 0.73 |
|  | Normal | 3.40(5.72) |  |
|  | lymphocytosis | 2.83(3.77) |  |

** P < 0.05 (significant) # Only one patient had leucocytosis*
